# Predicted DRD4 prefrontal gene expression moderates snack intake and stress perception in response to the environment in adolescents

**DOI:** 10.1371/journal.pone.0234601

**Published:** 2020-06-26

**Authors:** Andre Krumel Portella, Afroditi Papantoni, Catherine Paquet, Spencer Moore, Keri Shiels Rosch, Stewart Mostofsky, Richard S. Lee, Kimberly R. Smith, Robert Levitan, Patricia Pelufo Silveira, Susan Carnell, Laurette Dube

**Affiliations:** 1 Desautels Faculty of Management, McGill Center for the Convergence of Health and Economics, McGill University, Montreal, QC, Canada; 2 Postgraduate Program in Pediatrics, Universidade Federal de Ciencias da Saude de Porto Alegre, Porto Alegre, RS, Brasil; 3 Department of Psychiatry and Behavioral Sciences, Division of Child and Adolescent Psychiatry, Johns Hopkins University School of Medicine, Baltimore, MD, United States of America; 4 Australian Centre for Precision Health, School of Health Sciences, University of South Australia, Adelaide, South Australia, Australia; 5 Department of Health Promotion, Education, and Behavior, Arnold School of Public Health, University of South Carolina, Columbia, SC, United States of America; 6 Center for Neurodevelopmental and Imaging Research and Department of Neuropsychology, Kennedy Krieger Institute, Baltimore, MD, United States of America; 7 Department of Neurology, Johns Hopkins University School of Medicine, Baltimore, MD, United States of America; 8 Centre for Addition and Mental Health (CAMH), Toronto, ON, Canada; 9 Department of Psychiatry, University of Toronto, Toronto, QC, Canada; 10 Ludmer Centre for Neuroinformatics and Mental Health, Montreal, QC, Canada; 11 Department of Psychiatry, McGill University, Montreal, QC, Canada; Harvard Medical School, UNITED STATES

## Abstract

Body weight is substantially determined by eating behaviors, which are themselves driven by biological factors interacting with the environment. Previous studies in young children suggest that genetic influences on dopamine function may confer differential susceptibility to the environment in such a way that increases behavioral obesity risk in a lower socioeconomic status (SES) environment but decreases it in a higher SES environment. We aimed to test if this pattern of effect could also be observed in adolescence, another critical period for development in brain and behavior, using a novel measure of predicted expression of the dopamine receptor 4 (*DRD4*) gene in prefrontal cortex. In a sample of 76 adolescents (37 boys and 39 girls from Baltimore, Maryland/US, aged 14-18y), we estimated individual levels of *DRD4* gene expression (PredDRD4) in prefrontal cortex from individual genomic data using PrediXcan, and tested interactions with a composite SES score derived from their annual household income, maternal education, food insecurity, perceived resource availability, and receipt of public assistance. Primary outcomes were snack intake during a multi-item ad libitum meal test, and food-related impulsivity assessed using a food-adapted go/no-go task. A linear regression model adjusted for sex, BMI z-score, and genetic ethnicity demonstrated a PredDRD4 by composite SES score interaction for snack intake (p = 0.009), such that adolescents who had lower PredDRD4 levels exhibited greater snack intake in the lower SES group, but lesser snack intake in the higher SES group. Exploratory analysis revealed a similar pattern for scores on the Perceived Stress Scale (p = 0.001) such that the low PredDRD4 group reported higher stress in the lower SES group, but less stress in the higher SES group, suggesting that PredDRD4 may act in part by affecting perceptions of the environment. These results are consistent with a differential susceptibility model in which genes influencing environmental responsiveness interact with environments varying in obesogenicity to confer behavioral obesity risk in a less favorable environment, but behavioral obesity protection in a favorable one.

## Introduction

We are amidst an obesity epidemic [1] but not everyone becomes obese [2]. Part of this variability is attributable to environmental factors. For example, we know that individuals with lower socioeconomic status (SES), are generally more prone to obesity [3, 4], but there is still great heterogeneity in how individuals respond to the distinct environmental conditions that promote obesity. Some of this variation is likely due to genetic and related downstream biological factors that influence behavioral responses to the food environment [5–8]. A potential biological moderator of environmental influences is the mesocorticolimbic dopamine system. Dopamine is known to play a role in modulating general perceptual sensitivity, where extensive dopaminergic innervation of brain regulatory systems in ascending limbic-frontal circuits as well as descending and reciprocal striatal-thalamocortical circuits [9], may be especially sensitive to environmental changes [10].

In studies of young children, we have found evidence suggesting that genes influencing brain dopamine function may moderate environmental responses. For example, we previously demonstrated that 4-year old girls who were carriers of a hypofunctional polymorphism of the Dopamine Receptor 4 (7 tandem repeat of 48-base-pair region, in the third exon of the *DRD4* gene, or *DRD47R*)[11] showed greater sensitivity to environmental conditions such that they had a higher preference for fat when living in a low SES environment, but a *diminished* preference when living in a high SES environment as compared to non-carriers of the 7-repeat polymorphism[12]. In line with this finding, we have shown that carriers of this variant had increased likelihood of developing obesity at 4 years of age if exposed to lower levels of maternal sensitivity as toddlers [6]. Recently we have demonstrated that genetically regulated expression of the *DRD4* gene (predDRD4) in prefrontal cortex interacts with the postnatal environment to predict emotional eating in 4yo, and desire to drink in 5yo children in two independent cohorts[13]. The above results may be thought of as an extension of Belsky’s Differential Susceptibility Hypothesis [14, 15] into the obesity realm, helping to explain individual differences in food preferences and food choices in response to different environments[5]. According to the Differential Susceptibility Hypothesis, genes previously thought of as “vulnerability” genes are in fact developmental plasticity genes that promote greater individual responsiveness to *both* positive (e.g. healthy fetal environment, warm/sensitive maternal care, high socioeconomic status) and negative (e.g. altered fetal environment, low maternal sensitivity, low socioeconomic status) environmental contexts [15].

Adolescence is a critical period for obesity development [16–18], and for the development of brain systems serving reward seeking behaviors and cognitive control [19]–processes that may underpin excessive consumption of palatable high fat and high sugar “junk” foods. Consistent with the Differential Susceptibility Hypothesis, candidate plasticity genes (such as *DAT1*, *DRD2*, *DRD4 5HTTLPR*, *COMT* and *MAOA*) have been shown to moderate the link between parenting quality and male adolescent self-regulation and impulsivity [15]. However, the Differential Susceptibility Hypothesis has yet to be tested within a male and female adolescent population in the context of obesity and associated behavioral factors, the latter of which may be most informative for targeted prevention.

Traditional candidate gene and genome-wide association studies probing human brain and behavior responses to environment have been very useful to understand gene-environment relationships relevant to many complex behaviors. However, since genetic data reflects code that is present in all cells of an organism, it conveys limited information regarding the genetically-driven biological mechanisms that influence each cell, tissue and system and ultimately determine phenotypes of interest[20]. This organ/tissue specificity is especially important for the study of phenotypes resulting from the functions of specific brain areas. Therefore, the aim of this study was to use a novel genomic approach that imputes the gene expression of *DRD4* in prefrontal cortex using individual level genomic information[21] to evaluate how genetically-influenced prefrontal brain dopamine function interacts with environmental obesity risk (captured by SES) to influence indices of behavioral (multi-item ad libitum meal intake) and neurobehavioral (food go no go task performance) obesity risk in adolescents. Also, because eating behavior and adiposity can be influenced by stress [22, 23], and because plasticity genes may indirectly impact eating behavior by altering the general perception of the environment, we conducted an exploratory analysis of differential susceptibility using perceptions of environmental stress as a secondary outcome.

## Methods

### Participants and procedures

Participants were part of a larger study investigating the neurobehavioral basis of obesity and familial obesity risk. Adolescents and their mothers were recruited via flyers posted at the Johns Hopkins Hospital in Baltimore, MD and online advertisements. For adolescents, exclusion criteria included being outside our target age range of 14–18 years old, current diagnosis of a significant health problem (e.g. eating disorder, learning disability), use of medication affecting appetite and body weight (e.g. stimulants, anti-depressants), participation in a structured weight loss program, medical contraindications to MRI (e.g. metal implants), and food allergies. For mothers, exclusion criteria included current pregnancy, and excessive smoking, recreational drug use or alcohol intake. Adolescent-mother dyads were required to speak English fluently. The sample was balanced based on current weight status and familial obesity risk of the adolescents, resulting in 3 groups: i) a lean low-risk group (adolescent <85^th^ BMI centile, mother BMI <25), ii) a lean high-risk group (adolescent <85^th^ BMI centile, mother BMI >25), and iii) an overweight/obese group (adolescent >85^th^ BMI centile, no requirement for maternal weight group).

Potential participants completed an initial telephone screening and eligible participants were then tested in a fed and fasted condition (counterbalanced across subjects). For the fed condition, participants consumed 474 ml/480 kcal Boost c.3.30pm, completed an MRI scan c.4pm, then underwent a multi-item ad libitum buffet meal test c. 5pm. For the fasted condition, participants consumed 474 ml/0 kcal water before the MRI scan and meal test. During the initial consultation, a total of 98 adolescents were consented/assented (parental consent and child assent for <18 y, self-consent for 18y or over). Fifteen completed neither test day and were excluded from further analysis. Of the remaining 83 participants, we excluded adolescent-mother pairs with incomplete socioeconomic information or missing meal intake data, resulting in a final sample of 76 adolescent-mother pairs who completed the initial consultation and at least one of the test days (no data were imputed). Baseline characteristics are given in Table 1. Demographic characteristics (child age, child sex, child race, child BMI z-score, and household income) were similar between the entire consented (n = 98) and final (n = 76) samples. However, maternal education was significantly lower in the entire sample (n = 98) compared to the final sample (p = 0.015), i.e. 14.4% had less than high school education in the entire sample vs. 11.8% in the final sample. This study was approved by the Johns Hopkins University School of Medicine Institutional Review Board.

**Table 1:** Sample baseline characteristics (N = 76).

	Mean (or N)	SD (or %)
Age (years)	16.1	(1.2)
Female	39	(51.3)
BMI	24.2	(6.3)
BMI z-score	0.54	(1.23)
BMI percentile	63.1	(33.3)
**Weight Group**		
Lean	45	(59.2)
Overweight	13	(17.1)
Obese	18	(23.7)
**Familial Risk Group**		
Lean-LR	22	(28.9)
Lean-HR	23	(30.3)
Overweight	31	(40.8)
**Race**		
White	42	(55.3)
Black/African-American	25	(32.9)
Asian	2	(2.6)
More than one race	6	(7.9)
Other/Unknown	1	(1.3)
**Annual Household Income**		
0–49,999	26	(34.2)
50,000–79,999	20	(26.3)
80,000 or more	30	(39.5)
**Maternal Education Level**		
High school graduate or less	9	(11.8)
College or equivalent training	42	(55.3)
Post graduate	25	(32.9)
**Food Security**		
Yes	58	(76.3)

### Measures

#### Anthropometric measures

Body weight and fat percentage were assessed at the initial consultation using a SC-331S Total Body Composition Analyzer (TANITA Corp., Tokyo), which measures body weight and estimates fat percentage via Bio-Impedance Analysis. Height was assessed using a wall-mounted stadiometer after shoe removal. BMI values (kg/m^2^) were calculated, and BMI z scores and percentiles were derived for adolescents, based on Center for Disease Control (CDC) growth charts from 2000 [24]. For adolescents, those under the 85^th^ percentile were classified as normal-weight, those between the 85th and 95^th^ percentiles as overweight, and those at the 95th percentile or above as obese.

#### Food go/no-go task

To assess food-related impulsivity, a food go/no go task was administered. This task was adapted from an existing simple go/no go task[25]. Participants were instructed to press a button as quickly as possible in response to a picture of a low energy-density food (broccoli; ‘go’ trial) but to inhibit pressing the button in response to a picture of a high energy-density food (French fries or ice cream, depending on preference as stated on day of testing; ‘no-go’ trial). Stimuli were presented for 300 ms, followed by a fixation cross (1,500 ms). The task was divided into two 4-min 7 sec runs, each with 78 go and 26 no-go trials. ‘Go’ trials were presented in consecutive groups of 1–6, while ‘no-go’ trials never appeared more than twice in a row. Thus, ‘no-go’ stimuli were effectively jittered, with a varying number of preceding go stimuli. Each run began and ended with a 10-s rest period; four 10-s rest periods also occurred at irregular intervals during each run. Reaction times (RTs) were recorded during the entire trial length.

#### Multi-item ad-libitum buffet meal

To assess eating behavior in response to an environmental challenge, a multi-item ad libitum buffet meal was administered. Adolescents were brought into a room in which they were presented with an ad-libitum buffet meal, including three 12” pizzas cut in 12 slices [plain cheese (c. 790 g, 2009 kcal), vegetable (c. 940 g, 2050 kcal), pepperoni (c. 825 g, 2199 kcal)], hummus (c. 283 g, 700 kcal), ranch dressing (c. 224 g, 880 kcal), vanilla ice cream (c. 250 g, 530 kcal), chocolate chip cookies (c. 200 g, 970 kcal), fudge brownies (c. 350 g, 1200 kcal), M&Ms (c. 200 g, 1000 kcal), Ruffles potato chips (c. 200 g, 1143 kcal), Cheetos (c. 250 g, 1339 kcal), baby carrots (c. 250 g, 103 kcal), cherry tomatoes (c. 300 g, 55 kcal), celery sticks (c. 200 g, 32 kcal), grapes (c. 600 g, 414 kcal), water (20 fl oz, 0 kcal), regular Coke (20 fl oz, 240 kcal) and diet Coke (20 fl oz, 11 kcal). Adolescents were instructed to eat as much as they wanted. They were informed that they would be left alone for 30 minutes to eat but they could step out of the room if they were finished sooner or could request for extra time. To encourage ad libitum eating, participants were asked to “imagine this meal is your regular dinner” and “imagine not eating for 4–5 hours following this meal”. Each food was weighed separately prior to and following the meal (out of sight of the participant) to determine amount consumed. The ad libitum meal was preceded and followed by verbal appetite and stress ratings (e.g. hunger, fullness, stress) on 0–100 VAS scale.

For the purposes of the current report we focus on task and meal data from the fed condition only. Our rationale was that fasting could mask gene-by-environment interactions by inducing a homeostatic hunger state across all participants, thus reducing individual variation in dependent variables of interest. The neuroimaging data are the subject of a separate investigation and will be reported elsewhere.

#### SES composite score

To assess socioeconomic environment, we used a combination of variables collected as part of the larger study. Mothers completed a demographic questionnaire in which they reported their education level (Less than high school, High school graduate or GED, Post high school training other than college, Some college, Graduated from college, Post graduate), annual household income (10 categories between $0–19,000 and $100,000 or more), and their own and their child’s ethnicity. They also completed the Household Food Security Survey [26], a questionnaire assessing perceived resource availability [27], and the Project F-EAT survey, which contained a question assessing whether families receive public assistance [28]. Variables used for the composite score were: annual household income, maternal education, food insecurity, perceived resource availability, and receipt of public assistance (see below for details). These variables were selected a priori and represented the entirety of the variables assessing socioeconomic status in our sample.

#### Perceived stress

The Perceived Stress Scale (PSS) [29], a widely used psychological instrument assessing the degree to which situations in one’s life are perceived as stressful, was administered. The PSS is a 10-item questionnaire using 5-point ratings (0 = Never, 1 = Almost Never, 2 = Sometimes, 3 = Fairly Often, 4 = Very Often). Responses across the 10 PSS items were summed to create a total score such that higher values indicated more perceived stress in daily life (Cronbach’s alpha = 0.868).

#### Predicted prefrontal DRD4 expression

DNA for the adolescents was extracted from saliva samples, obtained using Oragene OG500 (DNAGenotek, Ottawa, Canada) saliva collection kits. Expression of *DRD4* in prefrontal brain regions was computed using a machine learning prediction method (PrediXcan) [21] that estimates tissue-specific gene expression based on individual-level genotype data. Genotyping for this cohort was conducted using the genome-wide Illumina Infinium Multi-Ethnic Global Array (MEGA), with clusters for the SNPs being defined using GenomeStudio version 2011.1 and GenTrain 1.0. Quality control on the genotyping calls has been previously described [30]. SNPs were verified for a genotyping rate ≥95% and no deviation from Hardy–Weinberg equilibrium (P < 0.001), and minor allele frequency ≥0.05, using PLINK [31, 32]. After quality control procedures and imputation, 1,767,525 SNPs were available for use in PrediXcan. Details on how the PrediXcan method creates prediction models of gene expression can be found elsewhere [33]. In brief, PrediXcan uses a machine learning approach to generate algorithms to estimate the genetically determined component of gene expression in specific brain regions at the individual level from the subject's genotype. The algorithm was built using a reference dataset from deceased human brain donors, being therefore tissue-specific. This reference dataset is composed of data from the GTEx project (version 7) [34], GEUVADIS [35] and DGN [36] containing both genotype and gene expression levels. The PrediXcan method was executed according to methods available in [21], and using GTEX version 7 frontal cortex eQTL model [34].

### Statistical analysis

#### Food go/no-go

Guidelines for exclusions as described in Patros et al [37] were followed. No participants needed to be excluded due to the proportion of go trials with RTs <200 ms exceeding .30, or omission error rate exceeding .50 (n = 0), indicating adequate attention during task presentation. Our primary outcome measure was number of commission errors for no-go stimuli (fGNG commission error), which reflects inhibitory control, with larger number of errors indicating poorer inhibitory control. Variables of interest were computed in MATLAB version 7.1 (The Mathworks, Inc., Natick, MA).

#### Multi-item ad-libitum buffet meal

For the analysis of meal intake, we created three primary variables: *snack intake* (included vanilla ice cream, chocolate chip cookies, fudge brownies, M&Ms, Ruffles potato chips, and Cheetos), *pizza intake* (included cheese, vegetable, and pepperoni pizzas), and *fruit and vegetable intake* (included baby carrots, cherry tomatoes, celery, and grapes) by summing the weights of each of the foods consumed within each group. In addition, nutrition facts labels for each food item were used to calculate total macronutrient (carbohydrate, sugar, fat, protein) intake for each participant.

#### SES composite score

To ensure that the 5 socioeconomic variables described above reflected the same underlying theoretical structure (SES) and to derive a composite score reflecting multiple dimensions of SES, we conducted a principal component analysis (PCA) [38–40] with Promax rotation. All variables loaded on a single component with loadings ranging from 0.678 to 0.891, supporting our choice to create a composite score. This component explained 59.1% of variance. Each of the socioeconomic variables was standardized and weighted by its factor loading to create an SES composite score in which a higher score indicates higher SES. A detailed description of the variables and PCA factor loadings is given in Table 2.

**Table 2:** Detailed variable description for SES composite score and PCA factor loadings.

	Type	Mean (SD)	Component 1 Loading
Annual Household Income	Ordinal	6.2 (3.2)	0.891
Maternal Education Level	Ordinal	4.6 (1.4)	0.772
Receiving Public Assistance (positive direction)	Dichotomous	0.8 (0.4)	0.793
Food Security	Dichotomous	0.8 (0.4)	0.690
Perceived Resource Availability	Continuous	12.1 (5.3)	0.678

KMO Measure of Sampling Adequacy = 0.796.

Bartlett’s Test of Sphericity: p<0.001.

### General statistical methods and differential susceptibility analysis

To test for differential susceptibility, linear regression models using continuous *DRD4* predicted gene expression values for the prefrontal cortex (Z variable), SES composite score (X variable) and the interaction term between these two variables (X*Z) were run for ad libitum intake (kcal) of snacks, pizza, and fruit and vegetables (primary outcomes), and ad libitum intake (grams) of carbohydrates, sugar, fat, and protein. To ensure that differences in ad libitum intake were not driven by differences in key demographic and anthropometric variables, the models were adjusted for BMI z-score, age, sex, and two principal components reflecting population stratification (PC1, PC2). These components were used as covariates to account for differences in ancestry and geographic origins in place of self-reported race, which can be inaccurate for genetic studies [41]. Following Roisman et al's recommendations [42], to ensure that any observed differential susceptibility effects are not an artifact of imposing a linear model on nonlinear relationships, additional linear regression models, including X^2^ and Z*X^2^ as predictors, were created to verify that neither of these two terms were statistically significant. This step was performed only for models with a significant X*Z interaction term. Post hoc analysis for the interaction terms included analysis of Proportion of Interaction (PoI) (i.e. the proportion of the total area represented in the interaction plots uniquely attributable to differential susceptibility), and proportion affected (PA) (i.e. the proportion of the population that is differentially affected by the moderator–Z variable) [42]. The regions of significance (RoS) analyses were conducted using a Web-based program developed by Fraley (http://www.yourpersonality.net/interaction). Preliminary analysis showed no interaction with sex, therefore in the main analysis boys and girls were analyzed together. Data were analyzed using the Statistical Package for the Social Sciences (SPSS) version 25.0 software (SPSS Inc., Chicago, IL, USA) and R software [43–45]. Significance levels for all results were set at p < 0.05. Results were corrected for multiple comparisons across all the linear regression models with False Discovery Rate (FDR) correction, using the Benjamini–Hochberg method (threshold set at q = 0.15) [46].

#### Confirming differential susceptibility

Following Roisman et al. [42], to verify differential susceptibility, when the RoS analyses are performed to determine whether the moderator (Z variable) and the outcome variable are correlated at the low and high ends of the distribution of the predictor (X variable), results should be considered significant only within a certain range of interest that is ±2SD of the observed predictor variable. Additionally, the PoI index should be roughly within 0.40 and 0.60 and the PA index should be close to 0.50.

## Results

Descriptive statistics for baseline characteristics can be found in Table 1. Linear regression beta coefficients and significance levels for the effect of the predictor variables ([*DRD4* predicted gene expression], [SES composite score], [*DRD4* predicted gene expression*SES composite score]) on the outcome variables, caloric intake and macronutrient intake, are displayed in Tables 3 and 4. *DRD4* predicted gene expression and the SES composite score had no significant main effects on the outcome variables when investigated separately from their interaction term, with the exception of the ad-libitum fat intake, where low SES composite score was associated with increased fat intake (*β =* -0.258, p = 0.040) (see S1 Data).

**Table 3:** Linear regression analyses results for caloric intake.

Variables	Snack Intake			Pizza Intake			Fruits & Vegetables Intake		
R^2^ (p-ANOVA)	0.241 (p = 0.017)			0.226 (p = 0.027)			0.229 (p = 0.885)		
	β	P	P_FDR_	β	P	P_FDR_	β	P	P_FDR_
*DRD4* predicted expression (Z)	0.049	0.680	0.868	-0.029	0.810	0.868	0.097	0.463	0.847
SES composite score (X)	0.125	0.438	0.847	-0.037	0.818	0.868	0.004	0.981	0.981
Z*X	0.407^¶^	**0.009**	**0.083**	0.051	0.739	0.868	0.077	0.653	0.868
Age	-0.118	0.303	0.747	0.171	0.142	0.506	-0.052	0.682	0.868
Sex	-0.297	**0.011**	**0.089**	-0.456	**<0.001**	**0.003**	0.043	0.737	0.868
BMI z-score	0.058	0.606	0.868	0.192	0.092	0.391	0.059	0.638	0.868
PC1	0.186	0.163	0.533	-0.053	0.693	0.868	-0.144	0.332	0.747
PC2	-0.129	0.258	0.743	-0.046	0.685	0.868	-0.126	0.322	0.747

PC1: Principal Component 1 for population stratification; PC2: Principal Component 2 for population stratification.

^¶^ Effect size attributable to interaction, R-Square change = 0.084 (p=0.009 for R-Square change). FDR threshold set at q=0.15

**Table 4:** Linear regression analyses results for macronutrient intake (grams).

Variables	Carbohydrates			Sugar			Fat			Protein		
R^2^ (p-ANOVA)	0.300 (p=0.002)			0.236 (p=0.020)			0.313 (p=0.001)			0.248 (p=0.013)		
	β	P	P_FDR_	β	P	P_FDR_	β	P	P_FDR_	β	P	P_FDR_
*DRD4* predicted expression (Z)	-0.005	0.967	0.	-0.037	0.756	0.868	0.	0.	0.868	0.028	0.811	0.868
SES composite score (X)	0.088	0.569	0.868	0.176	0.	0.747	-0.097	0.529	0.868	-0.024	0.882	0.907
Z*X	0.295	*0*.*047*a	0.251	0.318¶	**0.041**	0.251	0.249	0.089	0.391	0.087	0.567	0.868
Age	0.035	0.751	0.868	-0.019	0.868	0.906	0.061	0.576	0.868	0.173	0.131	0.506
Sex	-0.499	**<0.001**	**0.001**	-0.388	**0.001**	**0.014**	-0.487	**<0.001**	**0.001**	-0.479	**<0.001**	**0.002**
BMI z-score	0.218	**0.046**	0.251	0.166	0.142	0.506	0.206	0.056	0.268	0.222	**0.049**	0.251
PC1	0.103	0.418	0.837	0.193	0.148	0.506	0.038	0.764	0.868	-0.	0.	0.868
PC2	-0.101	0.352	0.768	-0.083	0.468	0.847	-0.	0.307	0.747	-0.082	0.	0.868

PC1: Principal Component 1 for population stratification; PC2: Principal Component 2 for population stratification. ^¶^ Effect size attributable to interaction, R-Square change = 0.084 (p=0.009 for R-Square change). FDR threshold set at q=0.15

PC1: Principal Component 1 for population stratification; PC2: Principal Component 2 for population stratification;

**a**: Significance level drops to 0.906, when both nonlinear terms X^2^, Z*X^2^ are included in the model (X^2^: p=0.090, Z*X^2^: p=0.026), indicating a nonlinear relationship between the predictor (carbohydrate intake) and SES composite score.

^¶^ Effect size attributable to interaction, R-Square change = 0.091 (p=0.041 for R-Square change). FDR threshold set at q=0.15

**Fig 1:**
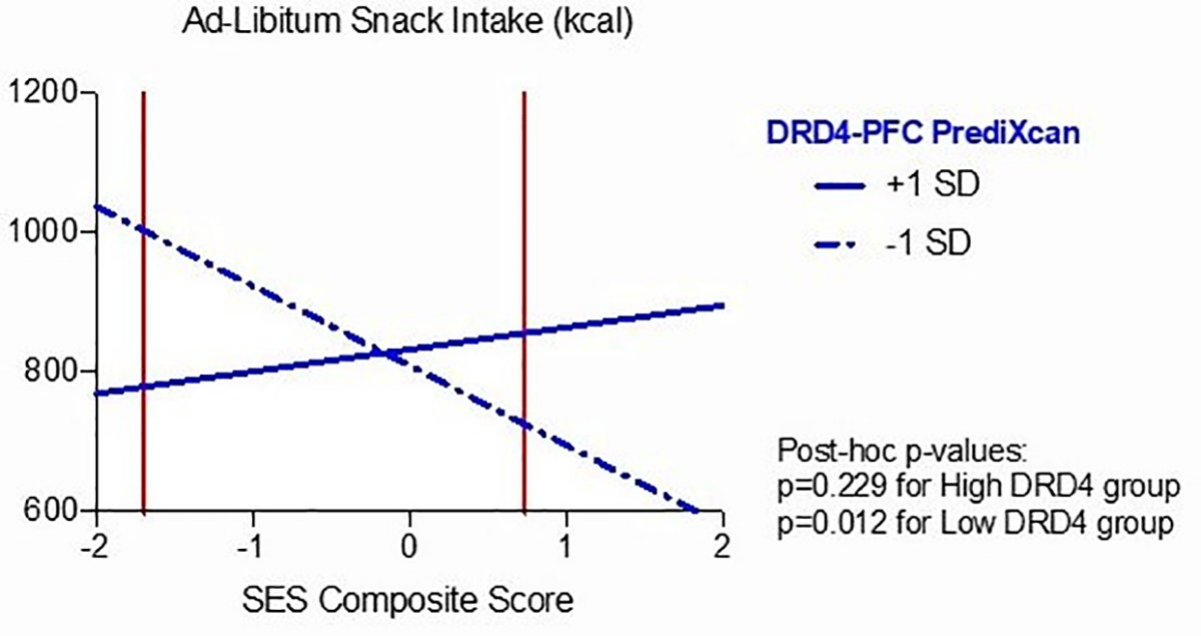
Effects of interaction between SES Composite Score and *DRD4* predicted gene expression on Ad-Libitum Snack Intake (kcal). The vertical lines depict the region of significance. The interaction occurs within the regions of significance providing evidence of differential susceptibility, such that lower predicted prefrontal (PFC) *DRD4* expression is associated with greater ad-libitum snack intake in adolescents with lower socioeconomic (SES) composite score.

*DRD4* predicted gene expression moderated the relationship between the SES composite score and ad-libitum snack intake (*β =* 0.407, p = 0.009). Furthermore, RoS analysis for the *DRD4* predicted gene expression effect revealed lower and upper bounds of significance within the observed predictor variable (for lower bound: SES composite score = -1.696; for higher bound: SES composite score = 0.736; simple slopes were significant outside this region). Finally, the proportion of interaction and the proportion affected/percentage above indices (PoI = 0.59, PA = 0.563) complied with a differential susceptibility model. Post hoc analysis for simple slopes for higher and lower predicted prefrontal *DRD4* gene expression levels (mean split) showed statistical significance only for the lower group (simple slope at Z = 0: -99.09, t(65) = 2.59, p = 0.012; simple slope at Z = 1: 52.25, t(65) = 1.21, p = 0.229), suggesting that only the low *DRD4* predicted expression group showed plasticity to the environmental conditions, such that lower SES composite score was associated with greater ad-libitum snack intake. Results can be seen in Fig 1.

**Fig 2:**
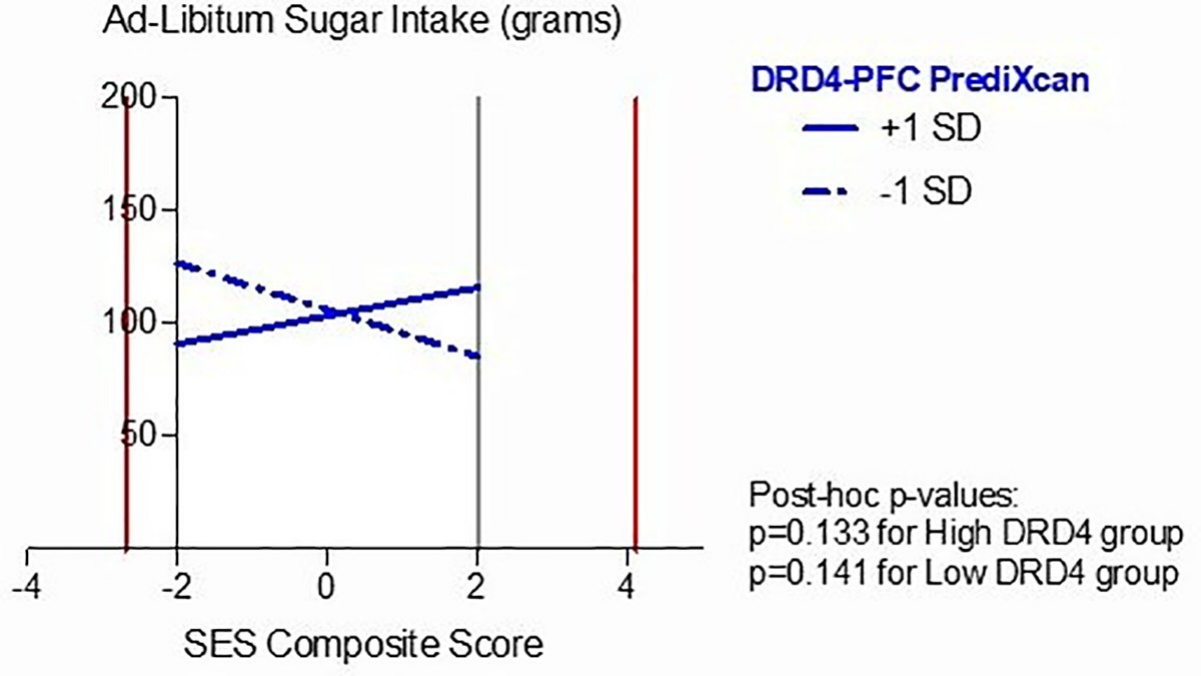
Effects of interaction between SES Composite Score and *DRD4* predicted gene expression on Ad-Libitum Sugar Intake (grams). The vertical lines depict the region of significance. Given that the vertical lines are outside the range of possible values for the SES composite score (range: [–2,2]), there is not sufficient evidence of differential susceptibility.

Additionally, *DRD4* predicted gene expression levels moderated the relationship between the SES composite score and ad-libitum sugar intake (*β =* 0.318, p = 0.041). However, the RoS analysis for *DRD4* predicted gene expression revealed lower and upper bounds of significance outside the observed predictor variable meaning that the significance of the difference can only be observed in extreme values of SES, beyond the observed values as shown in Fig 2 (for lower bound: SES composite score = -2.690; for higher bound: SES composite score = 4.113) and the proportion of interaction and the proportion affected/percentage above indices (PoI = 0.43; PA = 0.439) were not compatible with a differential susceptibility effect. Results can be seen in Fig 2.

*DRD4* predicted gene expression level did not moderate the relationship of the SES composite score with the other intake variables, or with commission errors on the food go/no go task (β = 0.130, p = 0.403).

Using a sub-sample of adolescents with available data (n = 71), we also conducted an exploratory analysis using Perceived Stress Scale scores. For initial exploration of relationships between stress and eating behavior, we used the SES composite score median to split our sample in two groups (higher SES with n = 36; lower SES with n = 35). Pearson’s correlations indicated that in the lower SES group, total PSS score positively correlated with ad-libitum sugar intake in the fed condition (r = 0.399, p = 0.022) and showed a positive trend with ad-libitum snack intake (r = 0.304, p = 0.086). In the higher SES group, total PSS score did not correlate with either sugar or snack intake (r = -0.081, p = 0.637; r = -0.047, p = 0.785, respectively).

**Fig 3:**
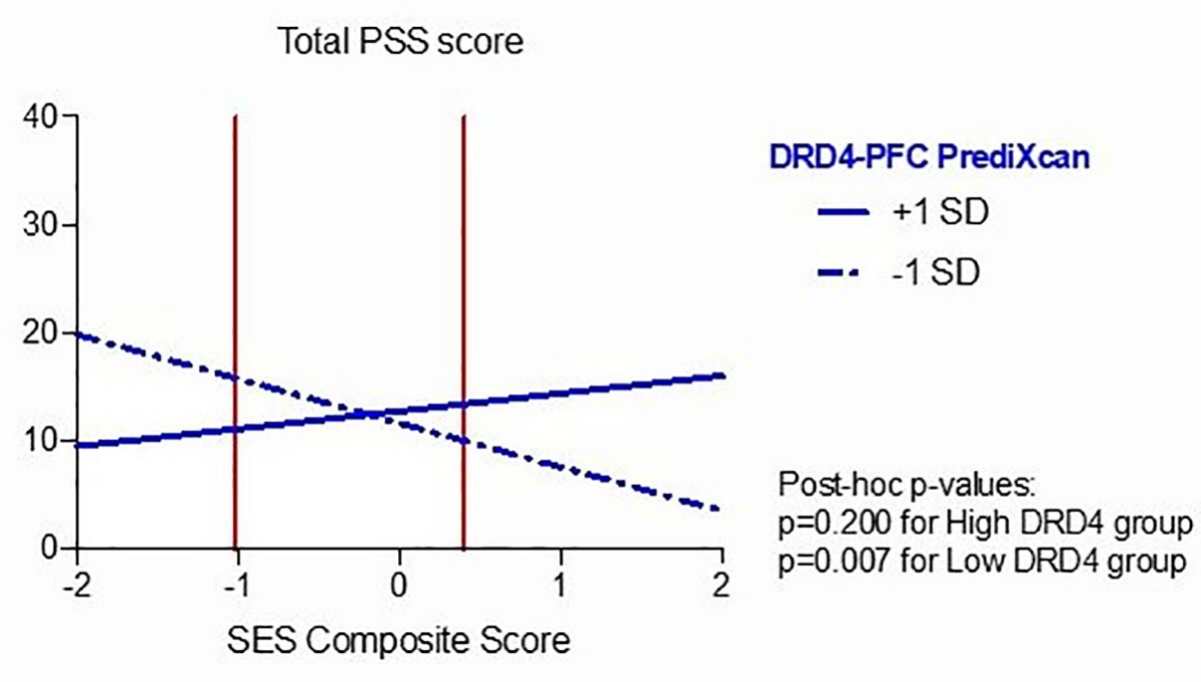
Effects of interaction between SES Composite Score and *DRD4* predicted gene expression on total PSS score. The vertical lines depict the region of significance. The interaction occurs within the regions of significance providing evidence of differential susceptibility, such that lower predicted prefrontal (PFC) *DRD4* expression is associated with greater Perceived Stress Scale (PSS) score in adolescents with lower socioeconomic (SES) composite score.

Subsequently, using the differential susceptibility framework method described above, we found that *DRD4* predicted gene expression moderated the relationship between SES composite score and PSS score (*β =* 0.552, p = 0.001). As for the eating behavior results described above, RoS for *DRD4* predicted gene expression revealed lower and upper bounds of significance within the observed predictor variable (for lower bound: SES composite score = -1.024; for higher bound: SES composite score = 0.397; simple slopes were significant outside this region), and the proportion of interaction and the proportion affected/percentage above indices (PoI = 0.60, PA = 0.578) complied with prototypical differential susceptibility. Post hoc analysis for simple slopes for the higher and lower predicted prefrontal *DRD4* gene expression levels showed statistical significance for the lower group only (simple slope at Z = 0: -3.15, t(62) = 2.79, p = 0.007; simple slope at Z = 1: 1.61, t(62) = 1.30, p = 0.200), suggesting that only the low *DRD4* group demonstrated plasticity to the environmental conditions, such that lower SES composite score was associated with greater PSS score. Results can be seen in Table 5 and Fig 3. Supporting the differential susceptibility framework, *DRD4* predicted gene expression and SES composite score had no main effect on PSS score when investigated separately from their interaction term (see S1 Data).

**Table 5:** Linear regression analysis results for Perceived Stress Scale (PSS) score.

Variables	PSS		
R^2^ (p-ANOVA)	0.219 (p=0.042)		
	β	P	P_FDR_
*DRD4* predicted expression (Z)	0.084	0.492	0.863
SES composite score (X)	0.207	0.206	0.617
Z*X	0.552^¶^	**0.001**	**0.009**
Age	0.035	0.768	0.868
Sex	0.064	0.588	0.868
BMI z-score	0.037	0.752	0.868
PC1	-0.135	0.324	0.747
PC2	-0.054	0.649	0.868

PC1: Principal Component 1 for population stratification; PC2: Principal Component 2 for population stratification.

^¶^ Effect size attributable to interaction, R-Square change = 0.164 (p=0.001 for R-Square change). FDR threshold set at q=0.15

## Discussion

In the current study, we describe the moderating effect of genetically predicted prefrontal *DRD4* gene expression on responses to the environment such that low frontal cortex expression of *DRD4* was associated with a higher environmental response, whereas a higher expression of *DRD4* was associated with no significative response to the environment. This effect of differential plasticity in response to environmental variation was observed across two different domains, with one outcome representing an actual eating behavior relevant to obesity development (snack intake in a buffet meal challenge), and the other a subjective rating of how stressful an individual perceives situations in his or her life to be. Importantly, our findings pertain to adolescence. This life period is crucial for investigation, since adolescents with obesity are more likely to maintain this phenotype through to adulthood than adolescents of normal weight [47]. Adolescence is a period especially vulnerable to psychological comorbidity in relation to obesity [48, 49]. Adolescence is also a critical period for prefrontal cortex development, including its dopamine innervation [19, 50–52], making it a sensitive window for the effects we observed here.

Specifically, in terms of eating behavior, adolescents with lower predicted prefrontal *DRD4* expression showed greater snack intake at an ad libitum meal test if they were of lower socioeconomic status (SES). Neighborhoods inhabited by lower SES populations have been shown to exhibit higher availability of calorie-dense food choices and associated food cues[53, 54], and such environmental forces have been associated with the rising trends in overconsumption and associated obesity over recent decades [55, 56]. SES also serves as a more general proxy of the quality of the surrounding environment, capturing factors including stress exposure [57] and lower opportunities for physical activity [58], as well as poorer access to nutritional foods [59]. Our results are therefore consistent with a differential susceptibility model whereby individual variations in dopamine-mediated openness to the environment affect the likelihood of unfavorable responses (i.e. snack intake) to unfavorable conditions (i.e. low SES), and might therefore also determine responses to improvements in such conditions [60].

This study also confirms findings on environmental plasticity from our previous research using the same genomic methodology (predicted prefrontal DRD4 expression). In that paper, Barth et al [13] demonstrated differential susceptibility effects on eating behavior in two ethnically distinct cohorts of children [13]. The present study extends these findings to an older age group, and provides support for perceived stress as an additional feature showing modulation by this gene by environment interaction.

Notably, analysis of macronutrients consumed during the meal test revealed a tendency toward a similar interaction effect for sugar calories consumed, although this variable did not show a formal differential susceptibility effect. Nevertheless, this pattern of results provides further support for the possibility that that prefrontal dopamine function may be specifically affecting behaviors towards palatable food. This is in accordance with a wealth of literature implicating the dopamine system in addictive like eating behavior [61], behavioral risk for obesity [62] and obesity itself [63].

The modulation of environmental responsiveness by *DRD4* gene expression that we observed here is consistent with well-established general functions of *DRD4*. *DRD4* functionally produces inhibitory effects, and is expressed in brain regions playing a role in planning, executive function and reward [64]. The *DRD4* exon III VNTR polymorphism (hypofunctional polymorphism), for instance, has been implicated in both planning/executive function effects and in heightened susceptibility to environmental influences [15], as well as with reduced inhibitory effects on postsynaptic neurons [65–67]. Such effects could be due to differential modulation of signal-to-noise ratio in subpopulations of mPFC neurons [68–71]. For example, afferent signals may be amplified relative to spontaneous basal firing (noise), thus affecting the signal to noise ratio and therefore consequent behavioral responses [69].

Our findings also build on previous literature demonstrating the role of dopamine function on responsiveness to food cues. For example, functional magnetic resonance (fMRI) imaging in response to imagined intake of palatable foods shows that future increases in body mass can be predicted by weaker brain activation of specific brain areas, particularly in individuals carrying low functioning variants of dopamine receptor genes, such as the *DRD2 TaqIA A1* allele or the *DRD47R* allele [72, 73]. The *DRD47R* polymorphism has been associated with markedly decreased affinity for dopamine and impaired intracellular signaling in comparison to other exon III alleles [74]. Our group has studied the *DRD47R* allele in several disorders characterized by increased eating that are most prevalent in females [7, 75, 76]. *DRD47R* carriers also report significantly more craving for food in a cue-elicited food-craving test [77].

Both cortical and subcortical brain regions control cognitive and behavioral responses to food cues, and food intake [78–81]. The balance between involuntary stimulus-driven processes (bottom-up, in response to stimulus exposure) and reflective goal-driven processes (top-down, related to information processing and cognition) determine cognitive representations of the reward value of food cues, attentional responses to such cues, and impulsive behaviors [81, 82], with combined perturbation of these processes likely to underlie the predisposition to overeat [83]. Moreover, adolescence is a period associated with poor inhibitory control resulting from ongoing maturation of neural systems subserving these functions [50, 84]. Therefore, the extent to which people show enhanced selective attention to food cues in tasks such as a food go/no go (i.e. attentional bias) provides a neurobehaviorally-informed index of individual differences in reactivity to food reward, as determined by this integrated system. In the present study, we did not find any association or interaction between predicted prefrontal *DRD4* expression and SES composite score on food go/no go performance. This negative finding contrasts with predictions based on other studies which have shown that alterations of dopaminergic pathways can impact both sensitivity to reward and impulsivity [85–87], that can lead to poor decision-making processes and maladaptive behaviors such as altered eating behavior and addiction [88–90]. Impulsive subjects show an inability to refrain from a stimulus-driven action, as measured by response inhibition paradigms such as the go/no-go task[91] and impulsivity has been associated with functional polymorphisms of dopamine-related genes [92]. These negative results suggest that, in this sample, behavioral inhibitory control did not play a critical role in the response to environment [93–95].

In addition to effects on food intake, we here demonstrated that predicted prefrontal *DRD4* gene expression moderated the effects of SES on stress perception. Only the low *DRD4* group reported higher perceived stress. In contrast there was no effect of low SES on perceived stress in those with higher expression of *DRD4*, suggesting that higher expression may confer a higher resilience or what could be called a higher “internal buffering capacity” to environmental conditions [96]. We also demonstrated greater intake with higher perceived stress within lower SES participants. This exploratory result requires replication but is consistent with an overall alteration in environmental perception among individuals with blunted dopamine function with potential impacts on eating behavior, rather than a specific effect on eating behavior responses to the food environment. Our finding is consistent with literature in healthy adults demonstrating that dopamine function is associated with perceptual experiences including sensitivity to pain [97] and responsivity to acute psychosocial stressors [98]. Also, in accordance with our finding, pre-clinical studies have demonstrated that diminished presynaptic dopamine regulation and function in *DRD4* deficient mice can produce increased sensitivity to aversive [99] as well as reward cues [100]. Further, human studies also found that diminished dopamine inhibitory feedback in *DRD47R* carriers is linked to weaker physiological dopamine signaling compared to non-carriers [101]. Human imaging studies have also suggested that *DRD47R* variations might impact neural reactivity to both aversive as well as rewarding cues, with alcohol cue-triggered reactivity in the OFC, anterior cingulate cortex (ACC), and striatum being greater in *DRD47R* carriers [102], and *DRD47R* carriers showed greater neural responsivity to unpleasant images [103].

### Limitations

This study contained only a small proportion of very low-income individuals in this study sample. Nevertheless, we were able to observe interactions with SES here, arguing for the existence of effects even at higher income ranges. This is consistent with other research demonstrating health effects of SES that are not restricted to conditions of poverty but distributed across a whole range of wealth [104]. A further cause for caution in interpreting these results is that the generalizability of the PrediXcan method to non-Caucasian populations is unclear [105], necessitating further validation and replication work, although we were able to find similar effects using the same methodology in a cohort of predominantly Asian individuals[13, 105]. PrediXcan is an imputation technique with some limitations inherent to its method, which aims to predict only the genomic-related proportion of gene expression, not real expression or associated protein levels or functional activity. The real expression of DRD4, and its biological function, could still be subject to variation between subjects due to gene-environment interactions. Indeed the actual differential susceptibility phenotype could even be causally related to other indirect mechanisms, such as network polygenic effects, where DRD4 could work as a hub gene, or relate to genes adjacent to DRD4. The association between the predicted DRD4 and differential susceptibility phenotype we show here is, though, in line with findings in two different cohorts with heterogeneous populations in terms of age and ethnic background [13], and findings on the DRD4-7R polymorphism from previous literature[6, 12, 106–111]. Notwithstanding the limitations described, the results we present here are consistent with a nonlinear moderating effect of dopamine function on neural responses [112, 113], such that low predicted *DRD4* expression in prefrontal cortex was associated with a more plastic phenotype, conferring obesity risk in more adverse environments, and obesity protection in predominantly favorable environments.

## Supporting information

S1 Data (DOCX)

S2 Data (ZIP)

## References

[1] Caballero B. The global epidemic of obesity: an overview. Epidemiologic reviews. 2007;29(1):1–5. Epub 2007/06/16. 10.1093/epirev/mxm012 . 17569676

[2] Ball K, Abbott G, Cleland V, Timperio A, Thornton L, Mishra G, et al Resilience to obesity among socioeconomically disadvantaged women: the READI study. Int J Obes (Lond). 2012;36(6):855–65. Epub 2011/09/21. 10.1038/ijo.2011.183 . 21931326

[3] Mutunga M, Gallagher AM, Boreham C, Watkins DC, Murray LJ, Cran G, et al Socioeconomic differences in risk factors for obesity in adolescents in Northern Ireland. Int J Pediatr Obes. 2006;1(2):114–9. Epub 2007/10/02. 10.1080/17477160600569560 . 17907324

[4] Ball K, Crawford D. Socioeconomic status and weight change in adults: a review. Soc Sci Med. 2005;60(9):1987–2010. Epub 2005/03/04. 10.1016/j.socscimed.2004.08.056 . 15743649

[5] Dalle Molle R, Fatemi H, Dagher A, Levitan RD, Silveira PP, Dube L. Gene and environment interaction: Is the differential susceptibility hypothesis relevant for obesity? Neuroscience and biobehavioral reviews. 2017;73:326–39. Epub 2016/12/28. 10.1016/j.neubiorev.2016.12.028 28024828 PMC5283807

[6] Levitan RD, Jansen P, Wendland B, Tiemeier H, Jaddoe VW, Silveira PP, et al A DRD4 gene by maternal sensitivity interaction predicts risk for overweight or obesity in two independent cohorts of preschool children. J Child Psychol Psychiatry. 2017;58(2):180–8. Epub 2016/10/12. 10.1111/jcpp.12646 . 27726127

[7] Levitan RD, Masellis M, Basile VS, Lam RW, Kaplan AS, Davis C, et al The dopamine-4 receptor gene associated with binge eating and weight gain in women with seasonal affective disorder: an evolutionary perspective. Biol Psychiatry. 2004;56(9):665–9. Epub 2004/11/04. 10.1016/j.biopsych.2004.08.013 . 15522250

[8] Paquet C, de Montigny L, Labban A, Buckeridge D, Ma Y, Arora N, et al The moderating role of food cue sensitivity in the behavioral response of children to their neighborhood food environment: a cross-sectional study. The international journal of behavioral nutrition and physical activity. 2017;14(1):86 Epub 2017/07/07. 10.1186/s12966-017-0540-9 28679391 PMC5499022

[9] Nigg JT, Casey BJ. An integrative theory of attention-deficit/ hyperactivity disorder based on the cognitive and affective neurosciences. Dev Psychopathol. 2005;17(3):785–806. Epub 2005/11/03. 10.1017/S0954579405050376 . 16262992

[10] Arnsten AF, Goldman-Rakic PS. Noise stress impairs prefrontal cortical cognitive function in monkeys: evidence for a hyperdopaminergic mechanism. Arch Gen Psychiatry. 1998;55(4):362–8. Epub 1998/04/29. 10.1001/archpsyc.55.4.362 . 9554432

[11] Chang FM, Kidd JR, Livak KJ, Pakstis AJ, Kidd KK. The world-wide distribution of allele frequencies at the human dopamine D4 receptor locus. Hum Genet. 1996;98(1):91–101. Epub 1996/07/01. 10.1007/s004390050166 . 8682515

[12] Silveira PP, Gaudreau H, Atkinson L, Fleming AS, Sokolowski MB, Steiner M, et al Genetic Differential Susceptibility to Socioeconomic Status and Childhood Obesogenic Behavior: Why Targeted Prevention May Be the Best Societal Investment. JAMA pediatrics. 2016;170(4):359–64. Epub 2016/02/03. 10.1001/jamapediatrics.2015.4253 . 26832777

[13] Barth B, Bizarro L, Miguel PM, Dube L, Levitan R, O'Donnell K, et al Genetically predicted gene expression of prefrontal DRD4 gene and the differential susceptibility to childhood emotional eating in response to positive environment. Appetite. 2020;148:104594 Epub 2020/01/14. 10.1016/j.appet.2020.104594 . 31927071

[14] Belsky J. The Differential Susceptibility Hypothesis: Sensitivity to the Environment for Better and for Worse. JAMA pediatrics. 2016;170(4):321–2. Epub 2016/02/03. 10.1001/jamapediatrics.2015.4263 . 26831915

[15] Belsky J, Jonassaint C, Pluess M, Stanton M, Brummett B, Williams R. Vulnerability genes or plasticity genes? Mol Psychiatry. 2009;14(8):746–54. 10.1038/mp.2009.44 19455150 PMC2834322

[16] Dietz WH. Critical periods in childhood for the development of obesity. Am J Clin Nutr. 1994;59(5):955–9. Epub 1994/05/01. 10.1093/ajcn/59.5.955 . 8172099

[17] Alberga A, Sigal R, Goldfield G, Prud'Homme D, Kenny G. Overweight and obese teenagers: why is adolescence a critical period? Pediatric obesity. 2012;7(4):261–73. 10.1111/j.2047-6310.2011.00046.x 22461384

[18] Luking KR, Pagliaccio D, Luby JL, Barch DM. Reward Processing and Risk for Depression Across Development. Trends in cognitive sciences. 2016;20(6):456–68. Epub 2016/05/02. 10.1016/j.tics.2016.04.002 27131776 PMC4875800

[19] Paus T. Mapping brain maturation and cognitive development during adolescence. Trends in cognitive sciences. 2005;9(2):60–8. Epub 2005/01/26. 10.1016/j.tics.2004.12.008 . 15668098

[20] Adams J. Transcriptome: connecting the genome to gene function. Nat Educ. 2008;1(1):195.

[21] Gamazon ER, Wheeler HE, Shah KP, Mozaffari SV, Aquino-Michaels K, Carroll RJ, et al A gene-based association method for mapping traits using reference transcriptome data. Nat Genet. 2015;47(9):1091–8. Epub 2015/08/11. 10.1038/ng.3367 26258848 PMC4552594

[22] Leng G, Adan RAH, Belot M, Brunstrom JM, de Graaf K, Dickson SL, et al The determinants of food choice. Proc Nutr Soc. 2017;76(3):316–27. Epub 2016/12/03. 10.1017/S002966511600286X . 27903310

[23] Sinha R, Jastreboff AM. Stress as a common risk factor for obesity and addiction. Biol Psychiatry. 2013;73(9):827–35. Epub 2013/04/02. 10.1016/j.biopsych.2013.01.032 23541000 PMC3658316

[24] Kuczmarski RJ, Ogden CL, Guo SS, Grummer-Strawn LM, Flegal KM, Mei Z, et al 2000 CDC Growth Charts for the United States: methods and development. Vital and health statistics Series 11, Data from the national health survey. 2002;(246):1–190. Epub 2002/06/05. . 12043359

[25] Mostofsky SH, Schafer JGB, Abrams MT, Goldberg MC, Flower AA, Boyce A, et al fMRI evidence that the neural basis of response inhibition is task-dependent. Cognitive Brain Research. 2003;17(2):419–30. 10.1016/s0926-6410(03)00144-7 PubMed PMID: WOS:000186120200022. 12880912

[26] Hager ER, Quigg AM, Black MM, Coleman SM, Heeren T, Rose-Jacobs R, et al Development and validity of a 2-item screen to identify families at risk for food insecurity. Pediatrics. 2010;126(1):e26–32. Epub 2010/07/03. 10.1542/peds.2009-3146 . 20595453

[27] Griskevicius V, Delton AW, Robertson TE, Tybur JM. Environmental contingency in life history strategies: the influence of mortality and socioeconomic status on reproductive timing. J Pers Soc Psychol. 2011;100(2):241–54. Epub 2010/09/30. 10.1037/a0021082 20873933 PMC3556268

[28] Bruening M, MacLehose R, Loth K, Story M, Neumark-Sztainer D. Feeding a family in a recession: food insecurity among Minnesota parents. Am J Public Health. 2012;102(3):520–6. Epub 2012/03/07. 10.2105/AJPH.2011.300390 22390517 PMC3349989

[29] Cohen S, Kamarck T, Mermelstein R. A global measure of perceived stress. J Health Soc Behav. 1983;24(4):385–96. Epub 1983/12/01. . 6668417

[30] Qiu A, Anh TT, Li Y, Chen H, Rifkin-Graboi A, Broekman BF, et al Prenatal maternal depression alters amygdala functional connectivity in 6-month-old infants. Transl Psychiatry. 2015;5:e508 Epub 2015/02/18. 10.1038/tp.2015.3 25689569 PMC4445753

[31] Qiu A, Shen M, Buss C, Chong YS, Kwek K, Saw SM, et al Effects of Antenatal Maternal Depressive Symptoms and Socio-Economic Status on Neonatal Brain Development are Modulated by Genetic Risk. Cerebral cortex. 2017;27(5):3080–92. Epub 2017/03/24. 10.1093/cercor/bhx065 28334351 PMC6057508

[32] Purcell S, Neale B, Todd-Brown K, Thomas L, Ferreira MA, Bender D, et al PLINK: a tool set for whole-genome association and population-based linkage analyses. Am J Hum Genet. 2007;81(3):559–75. Epub 2007/08/19. 10.1086/519795 17701901 PMC1950838

[33] Gamazon ER, Wheeler HE, Shah KP, Mozaffari SV, Aquino-Michaels K, Carroll RJ, et al A gene-based association method for mapping traits using reference transcriptome data. Nature genetics. 2015;47(9):1091 10.1038/ng.3367 26258848 PMC4552594

[34] Lonsdale J, Thomas J, Salvatore M, Phillips R, Lo E, Shad S, et al The Genotype-Tissue Expression (GTEx) project. Nature Genetics. 2013;45(6):580–5. 10.1038/ng.2653 PubMed PMID: WOS:000319563900002. 23715323 PMC4010069

[35] Lappalainen T, Sammeth M, Friedlander MR, t Hoen PA, Monlong J, Rivas MA, et al Transcriptome and genome sequencing uncovers functional variation in humans. Nature. 2013;501(7468):506–11. Epub 2013/09/17. 10.1038/nature12531 24037378 PMC3918453

[36] Battle A, Mostafavi S, Zhu XW, Potash JB, Weissman MM, McCormick C, et al Characterizing the genetic basis of transcriptome diversity through RNA-sequencing of 922 individuals. Genome Research. 2014;24(1):14–24. 10.1101/gr.155192.113 PubMed PMID: WOS:000329163500002. 24092820 PMC3875855

[37] Patros CH, L. Sweeney K, Mahone EM, Mostofsky SH, Rosch KS. Greater delay discounting among girls, but not boys, with ADHD correlates with cognitive control. Child Neuropsychology. 2018;24(8):1026–46. 10.1080/09297049.2017.1359525 28768457 PMC5796876

[38] Yu Q, Daugherty AM, Anderson DM, Nishimura M, Brush D, Hardwick A, et al Socioeconomic status and hippocampal volume in children and young adults. Dev Sci. 2018;21(3):e12561 Epub 2017/05/04. 10.1111/desc.12561 28464381 PMC5668203

[39] Price AL, Patterson NJ, Plenge RM, Weinblatt ME, Shadick NA, Reich D. Principal components analysis corrects for stratification in genome-wide association studies. Nat Genet. 2006;38(8):904–9. Epub 2006/07/25. 10.1038/ng1847 . 16862161

[40] Patterson N, Price AL, Reich D. Population structure and eigenanalysis. PLoS Genet. 2006;2(12):e190 Epub 2006/12/30. 10.1371/journal.pgen.0020190 17194218 PMC1713260

[41] Hellwege JN, Keaton JM, Giri A, Gao X, Velez Edwards DR, Edwards TL. Population Stratification in Genetic Association Studies. Curr Protoc Hum Genet. 2017;95(1):1 22 1–1 3. Epub 2017/10/19. 10.1002/cphg.48 29044472 PMC6007879

[42] Roisman GI, Newman DA, Fraley RC, Haltigan JD, Groh AM, Haydon KC. Distinguishing differential susceptibility from diathesis-stress: recommendations for evaluating interaction effects. Dev Psychopathol. 2012;24(2):389–409. Epub 2012/05/09. 10.1017/S0954579412000065 . 22559121

[43] J.A. L. _jtools: Analysis and Presentation of Social Scientific Data_. R package version 1.1.1, <URL: https://cran.r-project.org/package = jtools>. 2018.

[44] Wickham H. ggplot2: Elegant Graphics for Data Analysis. Springer-Verlag, editor. New York 2016.

[45] Team. RDC. R: A language and environment for statistical computing. Vienna, Austria2008.

[46] Benjamini Y, Hochberg Y. Controlling the False Discovery Rate—a Practical and Powerful Approach to Multiple Testing. Journal of the Royal Statistical Society Series B-Statistical Methodology. 1995;57(1):289–300. 10.1111/j.2517-6161.1995.tb02031.x PubMed PMID: WOS:A1995QE45300017.

[47] Malina RM, Katzmarzyk PT. Validity of the body mass index as an indicator of the risk and presence of overweight in adolescents. Am J Clin Nutr. 1999;70(1):131s–6s. PubMed PMID: WOS:000081224900031. 10.1093/ajcn/70.1.131s 10419416

[48] Libbey HP, Story MT, Neumark-Sztainer DR, Boutelle KN. Teasing, disordered eating behaviors, and psychological morbidities among overweight adolescents. Obesity (Silver Spring). 2008;16 Suppl 2(S2):S24–9. Epub 2008/12/17. 10.1038/oby.2008.455 . 18978759

[49] Boutelle KN, Hannan P, Fulkerson JA, Crow SJ, Stice E. Obesity as a prospective predictor of depression in adolescent females. Health psychology: official journal of the Division of Health Psychology, American Psychological Association. 2010;29(3):293–8. Epub 2010/05/26. 10.1037/a0018645 20496983 PMC2877273

[50] Hoops D, Flores C. Making Dopamine Connections in Adolescence. Trends in neurosciences. 2017;40(12):709–19. Epub 2017/10/17. 10.1016/j.tins.2017.09.004 29032842 PMC5705341

[51] Staff RT, Murray AD, Ahearn TS, Mustafa N, Fox HC, Whalley LJ. Childhood socioeconomic status and adult brain size: childhood socioeconomic status influences adult hippocampal size. Ann Neurol. 2012;71(5):653–60. Epub 2012/04/24. 10.1002/ana.22631 . 22522480

[52] Sowell ER, Peterson BS, Kan E, Woods RP, Yoshii J, Bansal R, et al Sex differences in cortical thickness mapped in 176 healthy individuals between 7 and 87 years of age. Cerebral cortex. 2006;17(7):1550–60. 10.1093/cercor/bhl066 16945978 PMC2329809

[53] Walker RE, Keane CR, Burke JG. Disparities and access to healthy food in the United States: A review of food deserts literature. Health Place. 2010;16(5):876–84. Epub 2010/05/14. 10.1016/j.healthplace.2010.04.013 . 20462784

[54] Gee GC, Payne-Sturges DC. Environmental health disparities: a framework integrating psychosocial and environmental concepts. Environmental health perspectives. 2004;112(17):1645–53. Epub 2004/12/08. 10.1289/ehp.7074 15579407 PMC1253653

[55] Swinburn BA, Sacks G, Hall KD, McPherson K, Finegood DT, Moodie ML, et al The global obesity pandemic: shaped by global drivers and local environments. Lancet. 2011;378(9793):804–14. Epub 2011/08/30. 10.1016/S0140-6736(11)60813-1 . 21872749

[56] Paeratakul S, Ferdinand DP, Champagne CM, Ryan DH, Bray GA. Fast-food consumption among US adults and children: dietary and nutrient intake profile. J Am Diet Assoc. 2003;103(10):1332–8. Epub 2003/10/02. 10.1016/s0002-8223(03)01086-1 . 14520253

[57] Kim GR, Jee SH, Pikhart H. Role of allostatic load and health behaviours in explaining socioeconomic disparities in mortality: a structural equation modelling approach. J Epidemiol Community Health. 2018;72(6):545–51. Epub 2018/02/21. 10.1136/jech-2017-209131 . 29459378

[58] Hobbs M, Griffiths C, Green MA, Jordan H, Saunders J, McKenna J. Associations between the combined physical activity environment, socioeconomic status, and obesity: a cross-sectional study. Perspect Public Health. 2018;138(3):169–72. Epub 2017/12/28. 10.1177/1757913917748353 . 29281499

[59] Caldwell AE, Sayer RD. Evolutionary considerations on social status, eating behavior, and obesity. Appetite. 2019;132:238–48. Epub 2018/08/07. 10.1016/j.appet.2018.07.028 30078673 PMC7039671

[60] Boyce WT, Ellis BJ. Biological sensitivity to context: I. An evolutionary-developmental theory of the origins and functions of stress reactivity. Dev Psychopathol. 2005;17(2):271–301. Epub 2006/06/10. 10.1017/s0954579405050145 . 16761546

[61] Volkow ND, Wise RA, Baler R. The dopamine motive system: implications for drug and food addiction. Nature reviews Neuroscience. 2017;18(12):741–52. Epub 2017/11/17. 10.1038/nrn.2017.130 . 29142296

[62] Baik JH. Dopamine signaling in reward-related behaviors. Front Neural Circuits. 2013;7:152 Epub 2013/10/17. 10.3389/fncir.2013.00152 24130517 PMC3795306

[63] Wang GJ, Volkow ND, Logan J, Pappas NR, Wong CT, Zhu W, et al Brain dopamine and obesity. Lancet. 2001;357(9253):354–7. Epub 2001/02/24. 10.1016/s0140-6736(00)03643-6 . 11210998

[64] Gilsbach S, Neufang S, Scherag S, Vloet TD, Fink GR, Herpertz-Dahlmann B, et al Effects of the DRD4 genotype on neural networks associated with executive functions in children and adolescents. Dev Cogn Neurosci. 2012;2(4):417–27. Epub 2012/06/26. 10.1016/j.dcn.2012.05.001 22727763 PMC7005761

[65] Asghari V, Sanyal S, Buchwaldt S, Paterson A, Jovanovic V, Van Tol HH. Modulation of intracellular cyclic AMP levels by different human dopamine D4 receptor variants. Journal of neurochemistry. 1995;65(3):1157–65. 10.1046/j.1471-4159.1995.65031157.x 7643093

[66] Van Tol HH, Wu CM, Guan HC, Ohara K, Bunzow JR, Civelli O, et al Multiple dopamine D4 receptor variants in the human population. Nature. 1992;358(6382):149–52. Epub 1992/07/09. 10.1038/358149a0 . 1319557

[67] D’Souza UM, Russ C, Tahir E, Mill J, McGuffin P, Asherson PJ, et al Functional effects of a tandem duplication polymorphism in the 5′ flanking region of the DRD4 gene. Biol Psychiat. 2004;56(9):691–7. 10.1016/j.biopsych.2004.08.008 15522254

[68] Cohen JD, Braver TS, Brown JW. Computational perspectives on dopamine function in prefrontal cortex. Current opinion in neurobiology. 2002;12(2):223–9. Epub 2002/05/17. 10.1016/s0959-4388(02)00314-8 . 12015241

[69] Vander Weele CM, Siciliano CA, Matthews GA, Namburi P, Izadmehr EM, Espinel IC, et al Dopamine enhances signal-to-noise ratio in cortical-brainstem encoding of aversive stimuli. Nature. 2018;563(7731):397–401. Epub 2018/11/09. 10.1038/s41586-018-0682-1 30405240 PMC6645392

[70] Rolls ET, Loh M, Deco G, Winterer G. Computational models of schizophrenia and dopamine modulation in the prefrontal cortex. Nature reviews Neuroscience. 2008;9(9):696–709. Epub 2008/08/21. 10.1038/nrn2462 . 18714326

[71] Winterer G, Weinberger DR. Genes, dopamine and cortical signal-to-noise ratio in schizophrenia. Trends in neurosciences. 2004;27(11):683–90. Epub 2004/10/12. 10.1016/j.tins.2004.08.002 . 15474169

[72] Stice E, Yokum S, Bohon C, Marti N, Smolen A. Reward circuitry responsivity to food predicts future increases in body mass: moderating effects of DRD2 and DRD4. NeuroImage. 2010;50(4):1618–25. Epub 2010/02/02. 10.1016/j.neuroimage.2010.01.081 20116437 PMC3987805

[73] Stice E, Spoor S, Bohon C, Small DM. Relation between obesity and blunted striatal response to food is moderated by TaqIA A1 allele. Science. 2008;322(5900):449–52. Epub 2008/10/18. 10.1126/science.1161550 18927395 PMC2681095

[74] Asghari V, Sanyal S, Buchwaldt S, Paterson A, Jovanovic V, Van Tol HH. Modulation of intracellular cyclic AMP levels by different human dopamine D4 receptor variants. J Neurochem. 1995;65(3):1157–65. Epub 1995/09/01. 10.1046/j.1471-4159.1995.65031157.x . 7643093

[75] Kaplan AS, Levitan RD, Yilmaz Z, Davis C, Tharmalingam S, Kennedy JL. A DRD4/BDNF gene-gene interaction associated with maximum BMI in women with bulimia nervosa. The International journal of eating disorders. 2008;41(1):22–8. Epub 2007/10/09. 10.1002/eat.20474 . 17922530

[76] Levitan RD, Masellis M, Lam RW, Kaplan AS, Davis C, Tharmalingam S, et al A birth-season/DRD4 gene interaction predicts weight gain and obesity in women with seasonal affective disorder: A seasonal thrifty phenotype hypothesis. Neuropsychopharmacology: official publication of the American College of Neuropsychopharmacology. 2006;31(11):2498–503. Epub 2006/06/09. 10.1038/sj.npp.1301121 . 16760922

[77] Sobik L, Hutchison K, Craighead L. Cue-elicited craving for food: a fresh approach to the study of binge eating. Appetite. 2005;44(3):253–61. Epub 2005/05/07. 10.1016/j.appet.2004.12.001 . 15876472

[78] Beaver JD, Lawrence AD, van Ditzhuijzen J, Davis MH, Woods A, Calder AJ. Individual differences in reward drive predict neural responses to images of food. The Journal of neuroscience: the official journal of the Society for Neuroscience. 2006;26(19):5160–6. Epub 2006/05/12. 10.1523/JNEUROSCI.0350-06.2006 16687507 PMC6674259

[79] Berridge KC. Wanting and Liking: Observations from the Neuroscience and Psychology Laboratory. Inquiry (Oslo). 2009;52(4):378 Epub 2010/02/18. 10.1080/00201740903087359 20161627 PMC2813042

[80] Rolls ET. Taste, olfactory and food texture reward processing in the brain and the control of appetite. Proc Nutr Soc. 2012;71(4):488–501. Epub 2012/09/20. 10.1017/S0029665112000821 . 22989943

[81] Rolls ET. Taste, olfactory and food texture reward processing in the brain and obesity. Int J Obes (Lond). 2011;35(4):550–61. Epub 2010/08/04. 10.1038/ijo.2010.155 . 20680018

[82] Winstanley CA, Theobald DE, Cardinal RN, Robbins TW. Contrasting roles of basolateral amygdala and orbitofrontal cortex in impulsive choice. The Journal of neuroscience: the official journal of the Society for Neuroscience. 2004;24(20):4718–22. Epub 2004/05/21. 10.1523/JNEUROSCI.5606-03.2004 15152031 PMC6729470

[83] Stice E, Spoor S, Ng J, Zald DH. Relation of obesity to consummatory and anticipatory food reward. Physiology & behavior. 2009;97(5):551–60. Epub 2009/03/31. 10.1016/j.physbeh.2009.03.020 19328819 PMC2734415

[84] Tamm L, Menon V, Reiss AL. Maturation of brain function associated with response inhibition. Journal of the American Academy of Child and Adolescent Psychiatry. 2002;41(10):1231–8. Epub 2002/10/05. 10.1097/00004583-200210000-00013 . 12364845

[85] Bear MF, Connors BW, Paradiso MA. Neuroscience exploring the brain. 4th ed: Wolters Kluwer; 2016.

[86] Li D, Sham PC, Owen MJ, He L. Meta-analysis shows significant association between dopamine system genes and attention deficit hyperactivity disorder (ADHD). Human molecular genetics. 2006;15(14):2276–84. 10.1093/hmg/ddl152 16774975

[87] Dunlop BW, Nemeroff CB. The role of dopamine in the pathophysiology of depression. Archives of general psychiatry. 2007;64(3):327–37. 10.1001/archpsyc.64.3.327 17339521

[88] Wilson GT. Eating disorders, obesity and addiction. European Eating Disorders Review. 2010;18(5):341–51. 10.1002/erv.1048 20821736

[89] Loxton NJ, Tipman RJ. Reward sensitivity and food addiction in women. Appetite. 2017;115:28–35. 10.1016/j.appet.2016.10.022 27756640

[90] Robbins TW, Clark L. Behavioral addictions. Current Opinion in Neurobiology. 2015;30:66–72. 10.1016/j.conb.2014.09.005 25262209

[91] Halperin JM, Wolf L, Greenblatt ER, Young G. Subtype Analysis of Commission Errors on the Continuous Performance-Test in Children. Developmental neuropsychology. 1991;7(2):207–17. 10.1080/87565649109540488 PubMed PMID: WOS:A1991FW52700007.

[92] Congdon E, Lesch KP, Canli T. Analysis of DRD4 and DAT polymorphisms and behavioral inhibition in healthy adults: implications for impulsivity. American journal of medical genetics Part B, Neuropsychiatric genetics: the official publication of the International Society of Psychiatric Genetics. 2008;147B(1):27–32. Epub 2007/05/26. 10.1002/ajmg.b.30557 . 17525955

[93] Diamond A. Executive functions. Annu Rev Psychol. 2013;64:135–68. Epub 2012/10/02. 10.1146/annurev-psych-113011-143750 23020641 PMC4084861

[94] Diamond A. Developmental Time Course in Human Infants and Infant Monkeys, and the Neural Bases of, Inhibitory Control in Reachinga. Annals of the New York Academy of Sciences. 1990;608(1):637–76. 2075965 10.1111/j.1749-6632.1990.tb48913.x

[95] Nigg JT. Annual Research Review: On the relations among self-regulation, self-control, executive functioning, effortful control, cognitive control, impulsivity, risk-taking, and inhibition for developmental psychopathology. J Child Psychol Psychiatry. 2017;58(4):361–83. Epub 2016/12/31. 10.1111/jcpp.12675 28035675 PMC5367959

[96] Russo SJ, Murrough JW, Han MH, Charney DS, Nestler EJ. Neurobiology of resilience. Nat Neurosci. 2012;15(11):1475–84. Epub 2012/10/16. 10.1038/nn.3234 23064380 PMC3580862

[97] Treister R, Pud D, Ebstein RP, Laiba E, Gershon E, Haddad M, et al Associations between polymorphisms in dopamine neurotransmitter pathway genes and pain response in healthy humans. Pain. 2009;147(1–3):187–93. Epub 2009/10/03. 10.1016/j.pain.2009.09.001 . 19796878

[98] White MJ, Lawford BR, Morris CP, Young RM. Interaction between DRD2 C957T polymorphism and an acute psychosocial stressor on reward-related behavioral impulsivity. Behav Genet. 2009;39(3):285–95. Epub 2009/01/17. 10.1007/s10519-008-9255-7 . 19148742

[99] Falzone TL, Gelman DM, Young JI, Grandy DK, Low MJ, Rubinstein M. Absence of dopamine D4 receptors results in enhanced reactivity to unconditioned, but not conditioned, fear. European Journal of Neuroscience. 2002;15(1):158–64. 10.1046/j.0953-816x.2001.01842.x 11860516

[100] Rubinstein M, Phillips TJ, Bunzow JR, Falzone TL, Dziewczapolski G, Zhang G, et al Mice lacking dopamine D4 receptors are supersensitive to ethanol, cocaine, and methamphetamine. Cell. 1997;90(6):991–1001. Epub 1997/10/10. 10.1016/s0092-8674(00)80365-7 . 9323127

[101] Nikolova YS, Ferrell RE, Manuck SB, Hariri AR. Multilocus genetic profile for dopamine signaling predicts ventral striatum reactivity. Neuropsychopharmacology: official publication of the American College of Neuropsychopharmacology. 2011;36(9):1940–7. Epub 2011/05/20. 10.1038/npp.2011.82 21593733 PMC3154113

[102] Filbey FM, Claus E, Audette AR, Niculescu M, Banich MT, Tanabe J, et al Exposure to the taste of alcohol elicits activation of the mesocorticolimbic neurocircuitry. Neuropsychopharmacology: official publication of the American College of Neuropsychopharmacology. 2008;33(6):1391–401. Epub 2007/07/27. 10.1038/sj.npp.1301513 17653109 PMC2856647

[103] Gehricke JG, Swanson JM, Duong S, Nguyen J, Wigal TL, Fallon J, et al Increased brain activity to unpleasant stimuli in individuals with the 7R allele of the DRD4 gene. Psychiatry research. 2015;231(1):58–63. Epub 2014/12/08. 10.1016/j.pscychresns.2014.10.021 25481571 PMC4272659

[104] Adler NE, Boyce T, Chesney MA, Cohen S, Folkman S, Kahn RL, et al Socioeconomic status and health. The challenge of the gradient. Am Psychol. 1994;49(1):15–24. Epub 1994/01/01. 10.1037//0003-066x.49.1.15 . 8122813

[105] Mikhaylova AV, Thornton TA. Accuracy of Gene Expression Prediction From Genotype Data With PrediXcan Varies Across and Within Continental Populations. Frontiers in Genetics. 2019;10:261. doi: ARTN 261 10.3389/fgene.2019.00261. PubMed PMID: WOS:000463434700001. 10.3389/fgene.2019.00261 31001318 PMC6456650

[106] Bakermans-Kranenburg MJ, van Ijzendoorn MH. Research Review: genetic vulnerability or differential susceptibility in child development: the case of attachment. J Child Psychol Psychiatry. 2007;48(12):1160–73. Epub 2007/12/21. 10.1111/j.1469-7610.2007.01801.x . 18093021

[107] Bakermans-Kranenburg MJ, Van IMH, Pijlman FT, Mesman J, Juffer F. Experimental evidence for differential susceptibility: dopamine D4 receptor polymorphism (DRD4 VNTR) moderates intervention effects on toddlers' externalizing behavior in a randomized controlled trial. Dev Psychol. 2008;44(1):293–300. Epub 2008/01/16. 10.1037/0012-1649.44.1.293 . 18194028

[108] Altink ME, Arias-Vasquez A, Franke B, Slaats-Willemse DI, Buschgens CJ, Rommelse NN, et al The dopamine receptor D4 7-repeat allele and prenatal smoking in ADHD-affected children and their unaffected siblings: no gene-environment interaction. J Child Psychol Psychiatry. 2008;49(10):1053–60. Epub 2008/11/20. 10.1111/j.1469-7610.2008.01998.x 19017022 PMC2870715

[109] Pluess M, Belsky J, Neuman RJ. Prenatal smoking and attention-deficit/hyperactivity disorder: DRD4-7R as a plasticity gene. Biol Psychiat. 2009;66(4):e5–e6. 10.1016/j.biopsych.2009.04.019 19500778

[110] Silveira PP, Portella AK, Kennedy JL, Gaudreau H, Davis C, Steiner M, et al Association between the seven-repeat allele of the dopamine-4 receptor gene (DRD4) and spontaneous food intake in pre-school children. Appetite. 2014;73:15–22. Epub 2013/10/25. 10.1016/j.appet.2013.10.004 24153108 PMC3872500

[111] Roth CL, Hinney A, Schur EA, Elfers CT, Reinehr T. Association analyses for dopamine receptor gene polymorphisms and weight status in a longitudinal analysis in obese children before and after lifestyle intervention. BMC Pediatr. 2013;13:197 Epub 2013/11/29. 10.1186/1471-2431-13-197 24283216 PMC4219494

[112] Gjedde A, Kumakura Y, Cumming P, Linnet J, Moller A. Inverted-U-shaped correlation between dopamine receptor availability in striatum and sensation seeking. Proc Natl Acad Sci U S A. 2010;107(8):3870–5. Epub 2010/02/06. 10.1073/pnas.0912319107 20133675 PMC2840468

[113] Romeo RD. Perspectives on stress resilience and adolescent neurobehavioral function. Neurobiol Stress. 2015;1:128–33. Epub 2015/01/01. 10.1016/j.ynstr.2014.11.001 27589663 PMC4721430

