# Supplementary material for: Predicted DRD4 prefrontal gene expression moderates snack intake and stress perception in response to the environment in adolescents
Source: PLoS One. 2020 Jun 26;15(6):e0234601. doi: 10.1371/journal.pone.0234601 (PMC7319347; doi:10.1371/journal.pone.0234601)
Supplement: S1 Data — (DOCX) [file pone.0234601.s001.docx]

**Predicted DRD4 prefrontal gene expression moderates snack intake and stress perception in response to the environment in adolescents.**

Supplemental Materials

**Supplementary Table 1a.** Linear regression analyses results for caloric intake, investigating main effects of *DRD4* predicted expression and SES composite score

| Variables | Snack Intake | | Pizza Intake | | Fruits & Vegetables Intake | |
| --- | --- | --- | --- | --- | --- | --- |
|  | β | P | β | P | β | P |
| *DRD4* predicted expression | -0.029 | 0.804 | -0.056 | -0.056 | 0.109 | 0.361 |
| SES composite score | -0.221 | 0.062 | -0.018 | -0.018 | 0.024 | 0.842 |

**Supplementary Table 1b.** Linear regression analyses results for macronutrient intake (grams), investigating main effects of *DRD4* predicted expression and SES composite score

| Variables | Carbohydrates | | Sugar | | Fat | | Protein | |
| --- | --- | --- | --- | --- | --- | --- | --- | --- |
|  | β | P | β | P | β | P | β | P |
| *DRD4* predicted expression | -0.084 | 0.479 | -0.124 | 0.296 | -0.022 | 0.850 | -0.011 | 0.928 |
| SES composite score | -0.134 | 0.259 | -0.114 | 0.333 | -0.255 | **0.030** | -0.038 | 0.753 |

**Supplementary Table 1c.** Linear regression analysis results for Perceived Stress Scale (PSS) score, investigating main effects of *DRD4* predicted expression and SES composite score

| Variables | PSS | |
| --- | --- | --- |
|  | β | P |
| *DRD4* predicted expression | 0.148 | 0.219 |
| SES composite score | -0.097 | 0.419 |

**
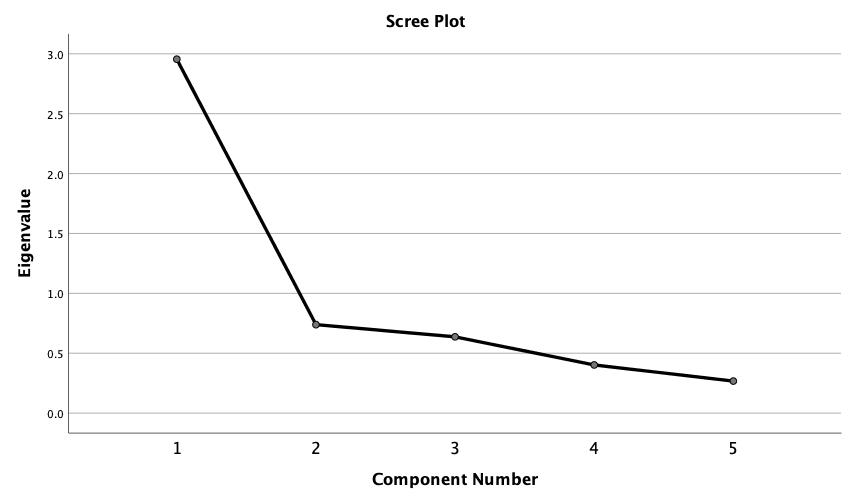
**

**Supplementary Figure 1**. Scree plot for the PCA performed on the 5 socioeconomic variables.
